# Understanding the Mortality Trends in Patients With Ventricular Arrhythmias and Cardiomyopathy: A 25‐Year Retrospective Analysis From 1999–2024

**DOI:** 10.1111/anec.70234

**Published:** 2026-10-12

**Authors:** Nosheen Pervaiz, Hamza Naveed, Muhammad Junaid Razzak, Shaheer Bin Shafiq, Muhammad Hasan, Sawera Gul, Maham Abbasi, Kamil Ahmad Kamil, Syed Ahmed Farhan

**Affiliations:** ^1^ Department of Internal Medicine Queen Elizabeth, Queen Mother Hospital Margate UK; ^2^ Department of Cardiology, Dow Medical College Dow University of Health Sciences Karachi Pakistan; ^3^ Department of Internal Medicine Capital Health Regional Medical Center Trenton New Jersey USA; ^4^ Chief, Department of Internal Medicine Nasrat Bashir Curative Hospital Kandahar Afghanistan; ^5^ Department of Internal Medicine McLaren Greater Lansing Hospital Lansing Michigan USA

**Keywords:** cardiomyopathy, differences, mortality trends, race, ventricular arrhythmias

## Abstract

**Background:**

Ventricular Arrhythmias (VA) associated with Cardiomyopathy (CM) continues to be the major cause of death. However, a national longitudinal study examining mortality trends from these conditions is still lacking. This study assessed mortality trends due to these conditions among U.S. adults aged ≥ 25 years from 1999 to 2024.

**Methods:**

An observational analysis of U.S. mortalities due to VA in patients with CM (ICD‐10 I47.2, I49.0 and I25.5, I42 respectively), was conducted using CDC WONDER. Adjusted Mortality Rates (AAMR) per 100,000 calculated and trends were assessed by calculating Average and Annual Percent Change (AAPC, APC), using joinpoint regression stratified by year, sex, race, and region.

**Results:**

From 1999 to 2024, 81,204 VA with CM‐related deaths occurred with the overall AAMR declined from 2.54 in 1999 to 1.01 in 2024 (AAPC: −3.55%; *p* < 0.000001). AAMRs decreased from 1999 to 2007 (APC: −7.93), remained stable till 2022 (APC: −0.66), and dropped by 2024 (APC: −6.87). Men exhibited greater AAMRs (2.42) than women (0.68). Racially, the AAMRs were most pronounced in Non‐Hispanic (NH) Blacks (1.97), followed by NH Whites (1.42), whereas Hispanics and NH Others experienced similar AAMRs of 0.97. Geographically, Delaware and the Western regions experienced the highest rates of mortality (2.2 and 1.53, respectively). Urban areas had higher AAMRs than rural settings (1.45 vs. 1.4)

**Conclusion:**

Although VA‐CM mortality has declined over the past two decades, differences by race, sex, and geography remain, underscoring the need for targeted healthcare strategies

## Graphical abstract

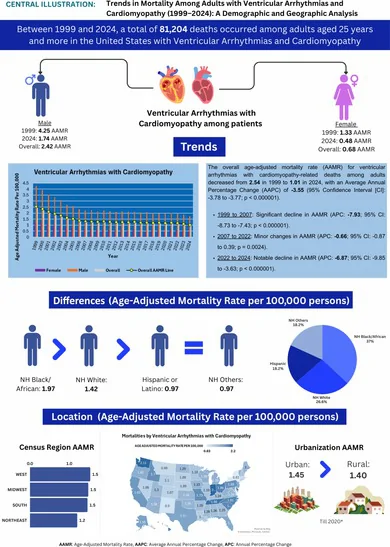

## 1 Introduction

Cardiomyopathy and ventricular arrhythmias (VAs) are two cardiac diseases. Their coexistence greatly increases the risk of negative health consequences, including sudden cardiac death (Priori et al. 2015). Cardiomyopathy is a group of diseases that impair the efficiency of the heart muscle in contracting blood (Brieler et al. 2017). This structural abnormality over time may lead to electrical instability of the heart (Mages et al. 2021), the perfect setting for fatal ventricular arrhythmias such as ventricular tachycardia (VT) and ventricular fibrillation (VF). In the United States, cardiomyopathy affects approximately 1 in 500 adults (CDC 2025a), but its burden is likely underestimated due to underdiagnosis and variable clinical presentation. VAs, meanwhile, contribute to more than 300,000 sudden cardiac deaths annually, highlighting their deadly potential when superimposed on weakened myocardium (Myerburg et al. 1997).

While cardiomyopathies and VAs are two distinct entities, their co‐occurrence carries significant clinical implications. The convergence of chronic myocardial injury (Crea 2022), interstitial fibrosis, and maladaptive electrical remodeling creates a vulnerable electrophysiological substrate, predisposing patients to sudden and potentially fatal arrhythmogenic events (Gomez et al. 2014). Individuals affected by both cardiomyopathy and VAs are associated with a more complicated clinical profile. They are hospitalized more often, are treated with device‐based therapy such as ICDs, and have higher mortality than the patient with either of these two entities (Zacharia et al. 2022).

Patho‐physiologically, cardiomyopathy and VAs interact with complex interplay. Structural remodeling and changes of the heart, chronic inflammation, neurohormonal disarray, and ischemia all contribute to arrhythmia susceptibility (Anand and Florea 2017; Taghdiri 2024; Grosman‐Rimon et al. 2019). Additionally, pharmacologic treatment can be particularly challenging for this subgroup of patients, with commonly used antiarrhythmic or heart failure drugs having proarrhythmic activity or adverse interactions when used in combination (Caballero et al. 2024). Optimal treatment strategy in these patients requires not only clinical expertise but also access to state‐of‐the‐art therapies, facilities not always globally available. In the presence of mounting evidence for such comorbidity, there remains a large gap in national‐level data reporting the epidemiological trend in death among both cardiomyopathy and ventricular arrhythmia patients. What evidence there is, nevertheless, addresses either condition independently, and important questions remain unanswered about the influence of this co‐occurrence on different sub‐groups within a population, for example, age, sex, race, ethnicity, or area.

To bridge this knowledge gap, the current study utilizes CDC WONDER mortality data for 1999 through 2024 to examine long‐term trends in death from ventricular arrhythmia cardiomyopathy in US adults aged 25 and older. Through stratification by demographic and geographic subgroups, we attempt to shed additional light on the extent to which this perilous pair of conditions causes overall cardiovascular mortality. Through such an examination, we attempt to shed insight on clinical practice, define populations at risk, and guide public health interventions to prevent preventable deaths.

## 2 Methods

### 2.1 Study Design and Data Source

We conducted a retrospective population‐based mortality study utilizing de‐identified death certificate data (1999–2024) from the Centers for Disease Control and Prevention Wide‐Ranging Online Data for Epidemiologic Research (CDC WONDER) database (CDC 2025b). The study population comprised U.S. residents aged ≥ 25 years whose death certificates listed ventricular arrhythmias (International Classification of Diseases, 10th Revision [ICD‐10] codes: I47.2 for ventricular tachycardia and I49.0 for ventricular fibrillation) concurrently with cardiomyopathy (ICD‐10 codes: I25.5 and I42) as underlying or contributing causes of death. These ICD‐10 codes align with validated clinical definitions for mortality ascertainment in cardiovascular epidemiology (Zipes et al. 2006). Records lacking complete demographic information were excluded from analysis. Consistent with federal regulations governing public de‐identified mortality data, this study was exempt from institutional review board oversight. Reporting adhered to the Strengthening Reporting of Observational Studies in Epidemiology (STROBE) guidelines (von Elm et al. 2007).

We attempted, where possible, to separate ischemic versus non‐ischemic cardiomyopathy using ICD‐10 subclassifications and cause‐of‐death descriptors; however, because of overlapping and unspecified coding on death certificates, accurate etiologic separation is limited and should be interpreted cautiously. Subtype counts are reported in Table S9.

### 2.2 Data Abstraction

Demographic and geographic variables were systematically extracted from CDC WONDER database, including year of death, age (≥ 25 years), sex (male/female), race/ethnicity categorized as Non‐Hispanic White, Non‐Hispanic Black, Hispanic, and Non‐Hispanic Other populations (including American Indian or Alaska Natives, Asian, Native Hawaiian or Other Pacific Islanders and other minority races), U.S. Census region (Northeast, Midwest, South, West), and urbanization status classified according to the 2013 National Center for Health Statistics scheme (urban: large central/fringe, medium, and small metropolitan; rural: micropolitan and noncore areas) (Aggarwal et al. 2021). Temporal context included documentation of the year of death (1999–2024) along with the recorded place of death. Independent data extraction was performed for each cardiomyopathy subgroup for subgroup‐specific analysis. Urban/rural classification information in CDC WONDER was available through 2020 for the scheme used; this availability constraint is reported and considered in subgroup analyses (see Table 2 and Table S8).

### 2.3 Statistical Analysis

Crude mortality rates per 100,000 persons were calculated annually by dividing ventricular arrhythmia‐cardiomyopathy associated deaths by the corresponding U.S. population estimates. Age‐adjusted mortality rates (AAMR) per 100,000 persons with 95% confidence intervals (CIs) were derived using direct standardization against the 2000 U.S. standard population (Anderson and Rosenberg 1998). Temporal trends were analyzed using the Joinpoint Regression Program (Version 5.1.0; National Cancer Institute) (Kim et al. 2000). Which implemented permutation tests to detect statistically significant inflection points across the 25‐year observation window. The analysis generated annual percent change (APC) metrics with 95% confidence intervals to quantify mortality trajectory alterations within piecewise linear segments, with statistical significance (*p* < 0.05) determined via two‐tailed *t*‐tests assessing deviation from zero slope. Stratified analyses examined mortality patterns across demographic and geographic subgroups, with pairwise APC comparisons assessing trend differences.

## 3 Results

Between 1999 and 2024, patients suffering from Ventricular Arrhythmias in patients with Cardiomyopathy accounted for a total of 81,204 deaths among adults aged 25 years and above in the United States (as shown in Table 1 and Table S1). These fatalities were distributed across various places of death, with the most occurring in medical facilities (78.79%), followed by decedent's home (15.27%), nursing homes/long‐term care facilities (3.38%), 1.55% at other locations, and 1.01% in hospice facilities (as shown in Table S2). The central illustration summarizing the study's characteristics and findings is presented in Figure 1.

**TABLE 1:** Demographic characteristics of deaths in the United States between 1999 and 2024.

Variable	Deaths (*n*)	Age adjusted mortality rate (AAMR) per 100,000
Overall population	81,204	1.43 (1.38–1.48)
Sex		
Male	59,834	2.42 (2.32–2.52)
Female	21,370	0.68 (0.63–0.73)
US Census Region		
Northeast	13,325	1.20 (1.10–1.31)
Midwest	18,825	1.48 (1.37–1.59)
South	30,461	1.46 (1.38–1.55)
West	18,593	1.53 (1.41–1.64)
Race/ethnicity		
Hispanic	4438	0.97 (0.82–1.13)
Non‐Hispanic Black	11,098	1.97 (1.78–2.16)
Non‐Hispanic White	62,820	1.42 (1.36–1.48)
Non‐Hispanic Others	2718	0.97 (0.78–1.19)
Urbanization		
Urban	57,261	1.45 (1.44–1.47)
Rural	11,556	1.40 (1.37–1.42)

**FIGURE 1:**
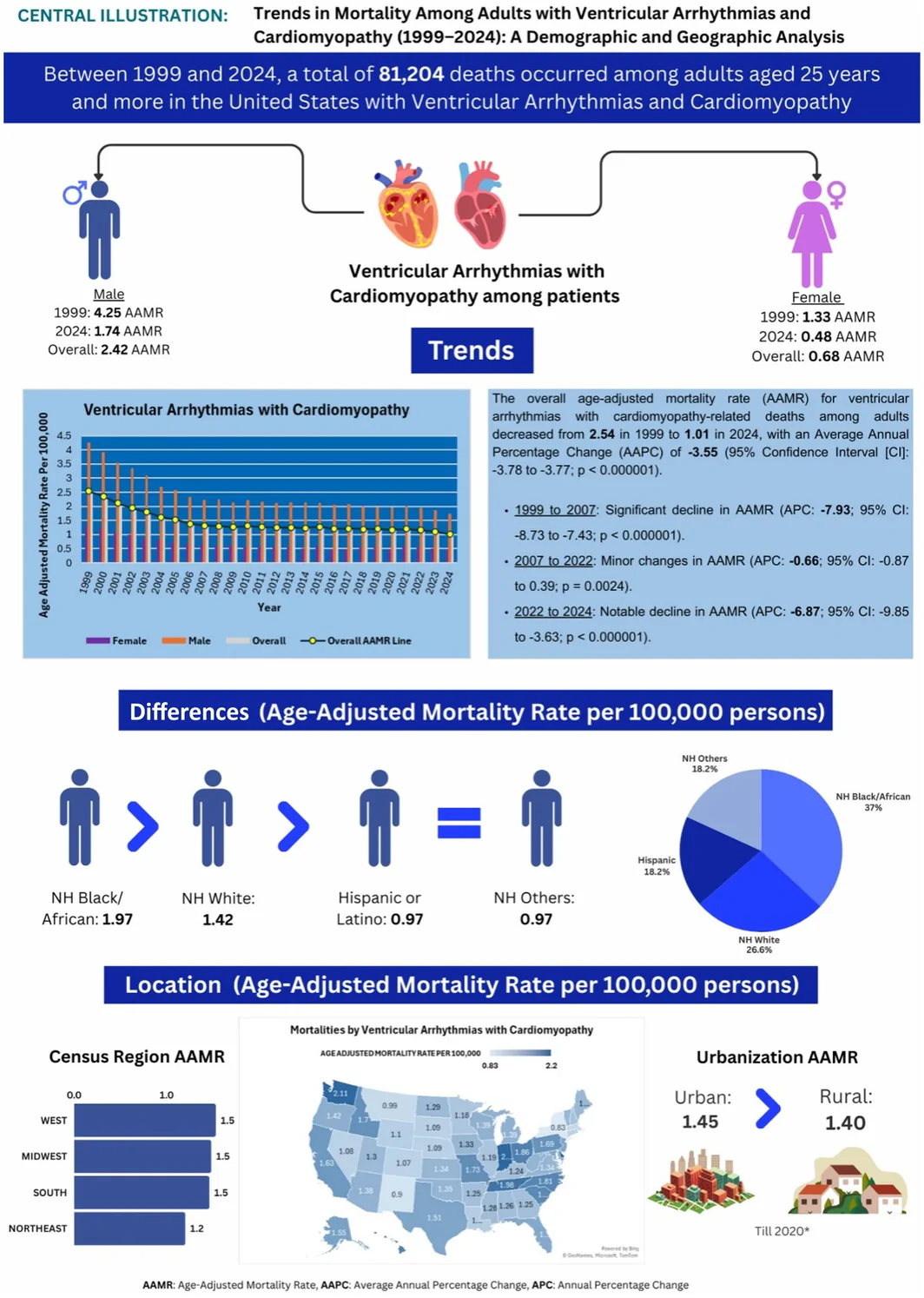
Central illustration depicting trends in demographics and differences in Ventricular Arrhythmias with Cardiomyopathy‐related Mortality among adults in the United States from 1999 to 2024.

### 3.1 Annual Trends

The Age‐Adjusted Mortality Rate (AAMR) for Ventricular Arrhythmias in patients with Cardiomyopathy‐related deaths among adults has shown a significant decrease from 2.54 in 1999 to 1.01 in 2024, with an Average Annual Percentage Change (AAPC) of −3.55 (95% Confidence Interval [CI]: −3.69 to −3.41). Notably, there was a significant decline in AAMR from 1999 to 2007 (APC: −7.93; 95% CI: −8.73 to −7.43), followed by minor changes from 2007 to 2022 (APC: −0.66; 95% CI: −0.87 to −0.39). Lastly, there was another notable decline in AAMR from 2022 to 2024 (APC: −6.87; 95% CI: −9.85 to −3.63) (as shown in Table 2 and Tables S3 and S4) (Figure 2).

**TABLE 2:** Annual percentage changes (APCs) and average annual percentage changes (AAPCs).

Variable	Trend segment	Year	APC (95% CI)	AAPC (95% CI)
Overall population	1	1999–2007	−7.93 (−8.73 to −7.43)	−3.55 (−3.69 to −3.41)
Overall population	2	2007–2022	−0.66 (−0.87 to −0.39)	−3.55 (−3.69 to −3.41)
Overall population	3	2022–2024	−6.87 (−9.85 to −3.63)	−3.55 (−3.69 to −3.41)
Sex				
Male	1	1999–2006	−8.54 (−10.16 to −7.58)	−3.51 (−3.81 to −3.23)
Male	2	2006–2022	−1.03 (−7.74 to 0.64)	−3.51 (−3.81 to −3.23)
Male	3	2022–2024	−5.08 (−8.86 to −1.16)	−3.51 (−3.81 to −3.23)
Female	1	1999–2008	−8.62 (−10.54 to −7.43)	−3.74 (−4.12 to −3.41)
Female	2	2008–2024	−0.88 (−1.58 to −0.11)	−3.74 (−4.12 to −3.41)
US Census Region				
Northeast	1	1999–2006	−9.39 (−11.68 to −7.63)	−3.06 (−3.42 to −2.72)
Northeast	2	2006–2024	−0.48 (−1.01 to 0.10)	−3.06 (−3.42 to −2.72)
Midwest	1	1999–2024	−2.52 (−3.49 to −1.59)	−2.52 (−3.49 to −1.59)
South	1	1999–2007	−8.15 (−9.30 to −7.26)	−3.42 (−3.61 to −3.24)
South	2	2007–2024	−1.12 (−1.43 to −0.77)	−3.42 (−3.61 to −3.24)
West	1	1999–2006	−8.08 (−11.48 to −6.19)	−3.51 (−3.89 to −3.11)
West	2	2006–2024	−1.67 (−2.24 to −1.04)	−3.51 (−3.89 to −3.11)
Race/ethnicity				
Hispanic	1	1999–2007	−10.24 (−19.74 to −6.60)	−4.05 (−4.81 to −3.12)
Hispanic	2	2007–2024	−1.00 (−2.24 to 0.90)	−4.05 (−4.81 to −3.12)
Non‐hispanic black	1	1999–2007	−6.67 (−11.18 to −4.85)	−2.77 (−3.19 to −2.31)
Non‐hispanic black	2	2007–2024	−0.88 (−1.57 to 0.00)	−2.77 (−3.19 to −2.31)
Non‐hispanic white	1	1999–2006	−8.74 (−10.12 to −7.67)	−3.28 (−3.48 to −3.07)
Non‐hispanic white	2	2006–2024	−1.07 (−1.40 to −0.69)	−3.28 (−3.48 to −3.07)
Non‐hispanic other	1	1999–2024	−2.22 (−3.29 to −0.92)	−2.22 (−3.29 to −0.92)
Urbanization				
Urban	1	1999–2007	−8.15 (−8.39 to −7.91)	−3.68 (−3.76 to −3.6)
Urban	2	2007–2020	−0.83 (−0.97 to −0.68)	−3.68 (−3.76 to −3.6)
Rural	1	1999–2006	−7.70 (−10.06 to −6.15)	−2.88 (−3.28 to 2.47)
Rural	2	2006–2020	−0.38 (−1.07 to 0.47)	−2.88 (−3.28 to 2.47)

**FIGURE 2:**
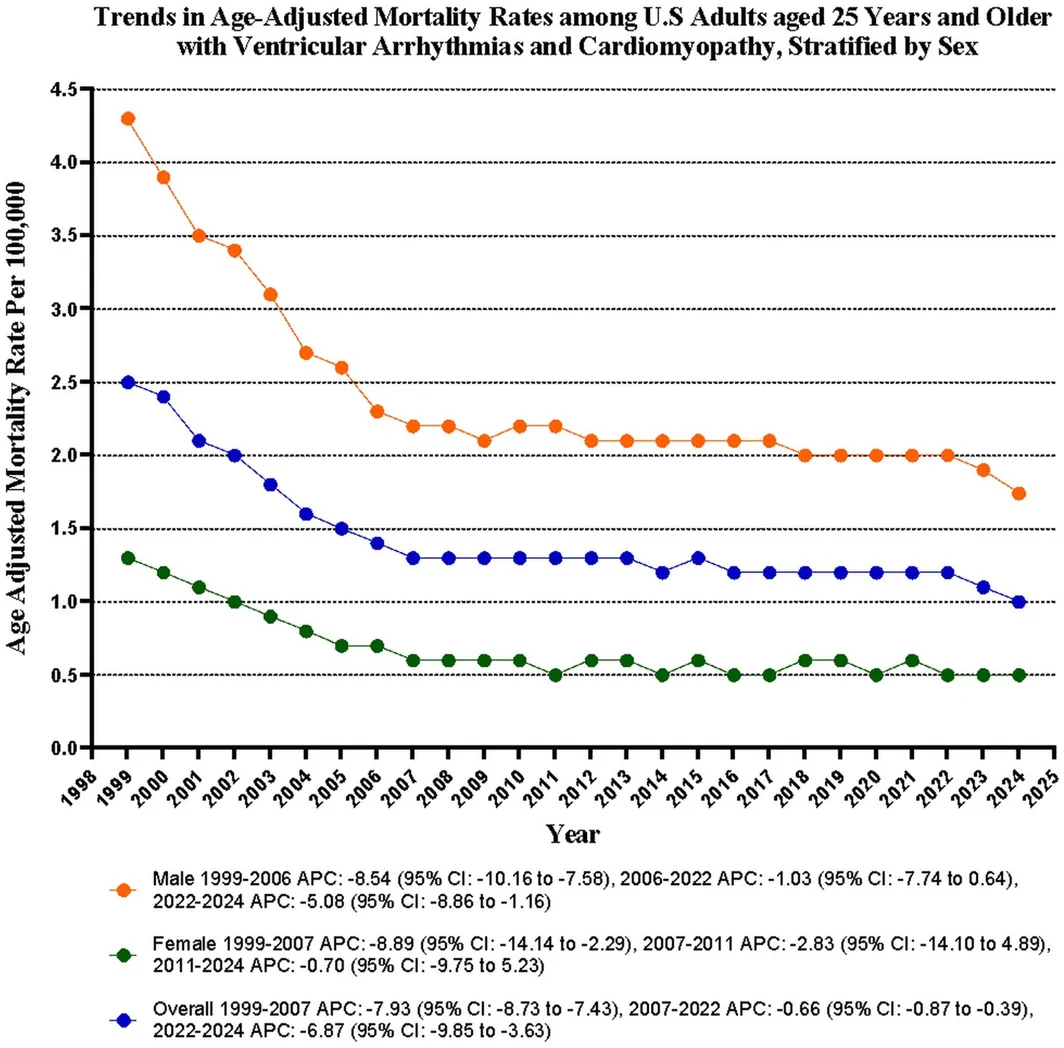
Sex‐Stratified Trend in Ventricular Arrhythmias in patients with Cardiomyopathy‐related AAMRs per 100,000, Among the adults of the United States, 1999–2024.

### 3.2 Sex

Throughout the study, adult men exhibited higher AAMRs than adult women (overall AAMR for men: 2.42, 95% CI: 2.32 to 2.52; for women: 0.68, 95% CI: 0.63 to 0.73). The AAMR for adult men showed variable trends, a significant decline was observed from 1999 to 2006 (APC: −8.54; 95% CI: −10.16 to −7.58), followed by a modest reduction from 2006 to 2022 (APC: −1.03; 95% CI: −7.74 to 0.64). Another substantial decline took place from 2022 to 2024 (APC: −5.08; 95% CI: −8.86 to −1.16). Although women also exhibited declining AAMR trends over the study period, the patterns differed slightly from those observed in men. A consistent and significant downward trend has been recorded from 1999 to 2008 (APC: −8.62), followed by a long period of a significant decline from 2008 to 2024 (APC: −0.88) (as shown in Table 2 and Tables S3 and S4) (Figure 2).

### 3.3 Race/Ethnicity

Significant variability in mortality rates was found among different racial/ethnic groups, with the highest mortality occurring in Non‐Hispanic (NH) White patients (62,820 deaths; 77.53%), followed by NH Black patients (11,098 deaths; 13.69%), Hispanic patients (4438 deaths; 5.48%), and least in NH Other populations (2718 deaths; 3.35%). The AAMRs were highest among NH Black or African Americans at 1.97, followed by NH Whites at 1.42, whereas Hispanic or Latinos and Non‐Hispanic Other populations showed similar AAMRs of 0.97.

All racial and ethnic groups had variable decreases in AAMRs from 1999 to 2024, with the most noticeable decrease observed in the Hispanic population. ([Hispanics: AAPC: −4.05]; [NH Whites: AAPC: −3.28]; [NH Black: AAPC: −2.77]; [NH Others: AAPC: −2.22]) (as shown in Table 2 and Tables S3–S5) (Figure 3).

**FIGURE 3:**
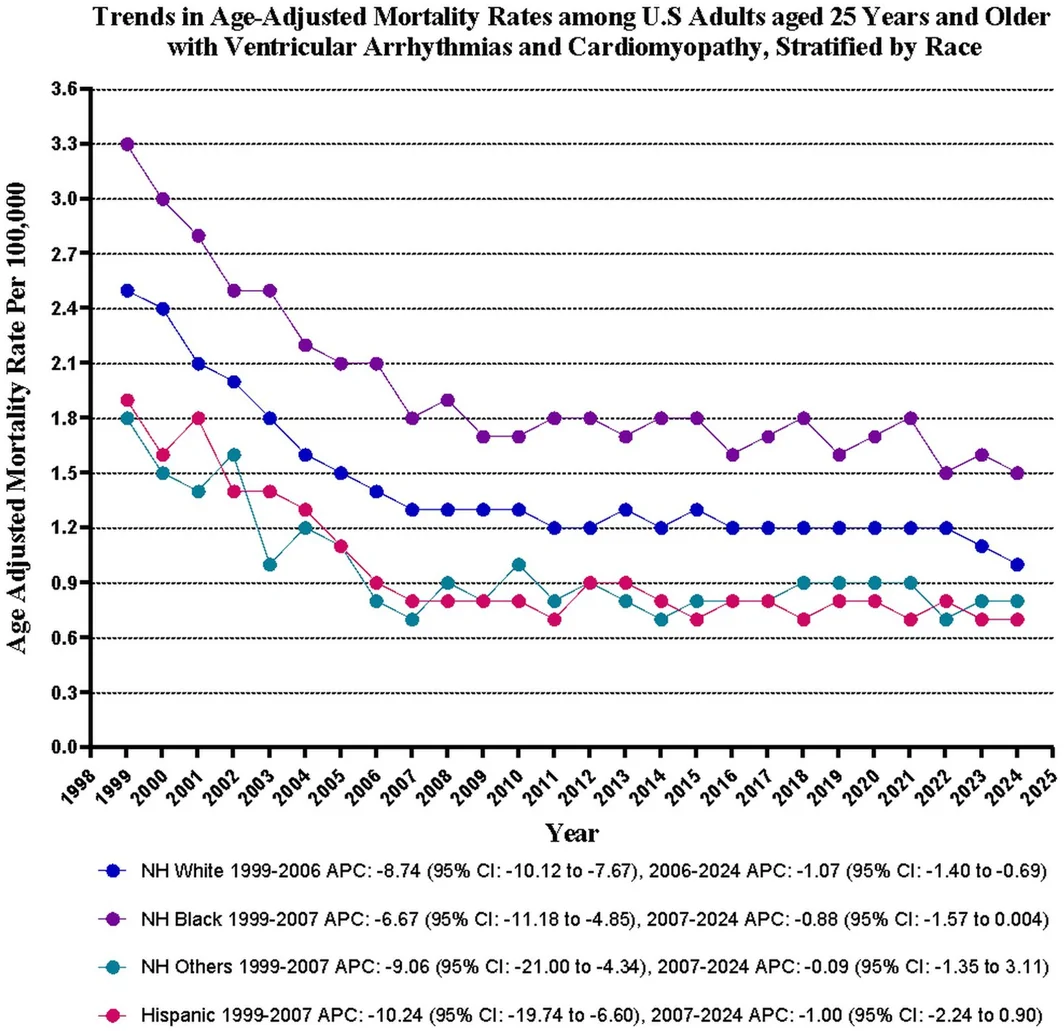
Race‐Stratified Trend in Ventricular Arrhythmias in patients with Cardiomyopathy‐related AAMRs per 100,000 Among the adults of the United States, 1999–2024.

### 3.4 Geographical Regions

Variations in AAMRs were observed among different states, with AAMRs ranging from as low as 0.83 (95% CI: 0.8 to 0.86) in New York up to 2.2 (95% CI: 1.95 to 2.44) in Delaware. States falling within the top 90th percentile included Delaware, Hawaii, Indiana, North Carolina, Ohio, and Tennessee, which had approximately 1.5 times higher AAMRs compared to states in the lower 10th percentile, which included Arizona, Connecticut, Colorado, Montana, Nevada, New Mexico, Nebraska, New York, and South Dakota (Table S6).

On average, over the study period, the highest mortality rates were observed in the Western (AAMR: 1.53), followed by Midwestern (AAMR: 1.48), South (AAMR: 1.46), and Northeast regions (AAMR: 1.2) (as shown in Table 2 and Tables S3–S7) (Figure 4).

**FIGURE 4:**
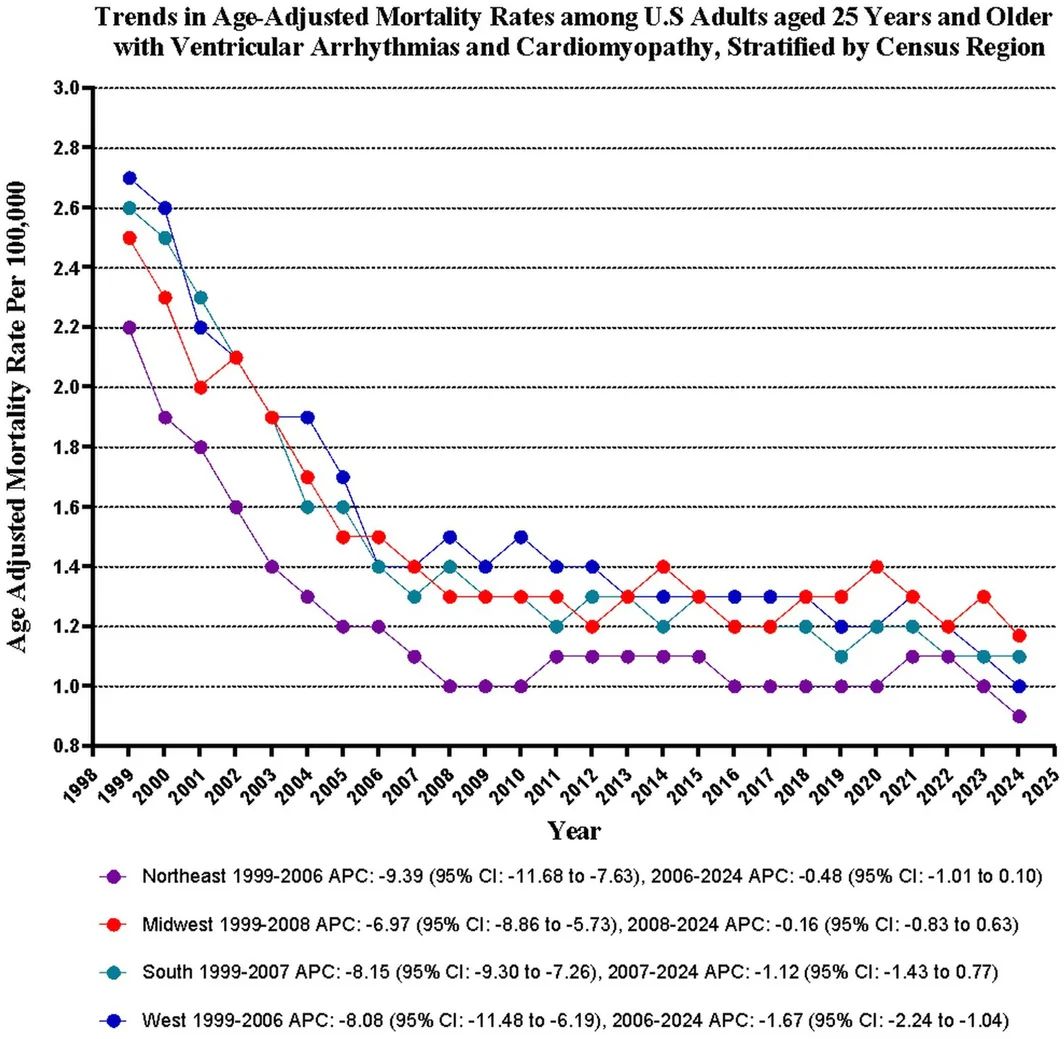
Census‐Stratified Trend in Ventricular Arrhythmias in patients with Cardiomyopathy‐related AAMRs per 100,000, Among the adults of the United States, 1999–2024.

Over the span of the study, metropolitan areas consistently displayed slightly higher AAMRs compared to nonmetropolitan areas, with overall AAMRs of 1.45 (95% CI: 1.44 to 1.47) and 1.4 (95% CI: 1.37 to 1.42) respectively. The AAMR of both metropolitan and nonmetropolitan areas experienced a decline from 1999 to 2020 [Metropolitan: AAPC: −3.68; Nonmetropolitan: AAPC: −2.88] (as shown in Table 2 and Tables S3–S8) (Figure 5).

**FIGURE 5:**
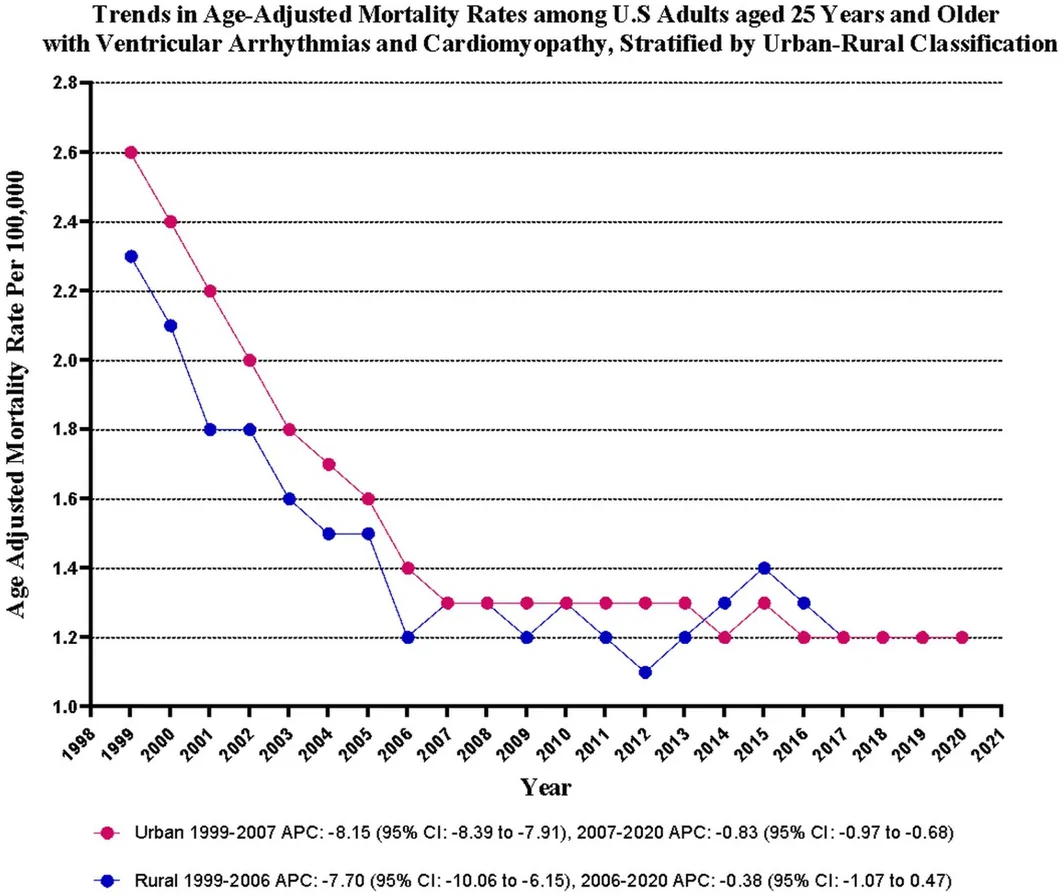
Urbanization‐Stratified Trend in Ventricular Arrhythmias in patients with Cardiomyopathy‐related AAMRs per 100,000, Among the adults of the United States, 1999–2024.

### 3.5 Subgroup Analysis

Subgroup analysis demonstrated important variation in ventricular arrhythmia (VA)–related mortalities across different cardiomyopathy subtypes from 1999 to 2024. Ischemic Cardiomyopathy accounted for the highest number of deaths (37,972), followed by mixed or unspecified Cardiomyopathy (33,173), and Dilated Cardiomyopathy (8352). Rest of the subgroups showed modest number of deaths with Alcoholic cardiomyopathy accounting for 607 deaths, (Obstructive hypertrophic cardiomyopathy: 163 deaths), (other hypertrophic cardiomyopathy: 823 deaths), (Endocardial fibroelastosis: 28 deaths), (Other restrictive cardiomyopathy: 110), (Cardiomyopathy due to drugs and external agents: 59), (Other cardiomyopathies: 135). No deaths were reported for endomyocardial eosinophilic disease (as shown in Table S9).

## 4 Discussion

Our study provides significant insights into the mortality associated with cardiomyopathy and ventricular arrhythmias across various demographic groups in the U.S. from 1999 to 2024. Throughout the study's duration, 81,204 deaths were attributed to diseases, with age‐adjusted mortality rates (AAMR) reflecting both the progress of therapeutic treatment and persistent challenges in preventive medicine and early detection. Though the overall AAMR has decreased over time, certain populations continue to experience higher mortality, pointing to the need for more specific public health strategies and improved access to specialized care for at‐risk groups; this difference highlights the ongoing need for tailored public health strategies and better access to specialized care.

The pathophysiology of these diseases is complex and involves myocardial fibrosis (Bittencourt et al. 2019), electrical remodeling, and ion channel dysfunction, all contributing to the genesis of ventricular arrhythmias and sudden cardiac death (SCD) in patients with dilated cardiomyopathy (DCM) (Gigli et al. 2024), hypertrophic cardiomyopathy (HCM), and arrhythmogenic right ventricular cardiomyopathy (ARVC) (Shah et al. 2024). These processes increase the likelihood of life‐threatening arrhythmias in hearts with both structural and electrical abnormalities, especially during periods of stress or ischemic events (Gopinathannair et al. 2015).

Throughout the evaluation period, men exhibited persistently greater AAMR compared to women, a trend that continued even when mortality rates for both sexes were falling. This noted difference in sex can be explained mostly by a multitude of factors such as the earlier development of the disease in males, genetic factors (Boussy et al. 2008), and differing behavior (Krisai et al. 2022). Males, particularly at younger ages, are found to present with ventricular arrhythmias (Maglia et al. 2022), for example, ventricular tachycardia (VT) and fibrillation, which are often risk factor related, for example, hypertension, coronary artery disease, and cardiac fibrosis (GYH et al. 2017). These conditions often culminate in sudden cardiac death (SCD), especially when occurring alongside dilated or ischemic cardiomyopathy (Polovina et al. 2023). Furthermore, high risk behaviors prevalent among men, such as smoking and alcohol abuse (Goh et al. 2024; Ritchie 2025), elevate the risk for developing ventricular arrhythmias, accelerating the onset of cardiomyopathy and SCD (Hartz et al. 1984; Cleveland Clinic 2025). Differences in sex‐specific mortality may also reflect differential access to diagnostic evaluation and device therapy (e.g., ICD implantation), variation in guideline‐directed therapy uptake, and potential under‐recognition of arrhythmic risk in women. These health system and treatment‐adoption factors could partly explain both the higher male AAMR and the changing temporal trends.

In contrast, arrhythmic conditions in women typically appear later in life (Zeitler et al. 2022), a fact partially due to the protective role of estrogen, which, besides other influences, enhances lipid metabolism and decreases the risk of atherosclerosis (Craig and Weber 2026). However, the withdrawal of estrogen's protective effect after menopause heightens vulnerability to heart failure and malignant arrhythmias (Lopez‐Pier et al. 2018). Women are also more likely to have heart failure with preserved ejection fraction (HFpEF) (Regitz‐Zagrosek 2020), a condition that may be underdiagnosed or misdiagnosed as non‐cardiac fatigue or other conditions. This highlights the gender‐specific challenges in diagnosing and managing these conditions. In addition, research has shown that interventions like implantable cardioverter‐defibrillator (ICD) insertion and antiarrhythmic medications are more effective in improving survival outcomes for men than for women (MacFadden et al. 2012), while women tend to receive these treatments less frequently (Curtis 2022). This can be explained by underdiagnosis of ventricular arrhythmias among women (Curtis and Narasimha 2012), in conjunction with a tendency for healthcare providers to postpone treatment relying on risk factors that do not adequately consider sex‐based differences in the pathophysiology of heart disease and symptom presentation (Villareal et al. 2001; Healy 1991).

Racial and ethnic differences have also been a significant characteristic of the epidemiology of cardiomyopathy and ventricular arrhythmias. Non‐Hispanic Blacks had the highest AAMR throughout the study period, with these conditions contributing disproportionately to cardiovascular mortality. This population is at greater risk for hypertension (Flack et al. 2007), which significantly increases the risk of arrhythmogenic cardiomyopathy and the occurrence of ventricular arrhythmias including ventricular tachycardia and fibrillation (Varvarousis et al. 2020). Hypertension results in myocardial fibrosis and cardiac remodeling that in turn promotes the generation of arrhythmias (Nguyen et al. 2017; Tomek and Bub 2017). Despite effective treatments, Black patients undergo undue delay in diagnosis and administration of treatment, ultimately resulting in poor outcomes. Underuse of ICDs (Tertulien et al. 2023) and antihypertensive therapy (Ferdinand et al. 2017) in this population exacerbates these health inequities underscoring the need for culturally appropriate healthcare interventions and increased access to specialist cardiac care (Singh et al. 2022). Moreover, the healthcare system's overreliance on general risk factors tends to overlook genetic factors that may predispose Black communities towards a greater risk of arrhythmias and sudden cardiac death (Banerjee et al. 2021). We emphasize that observed racial differences may reflect a combination of true differences in disease burden, differential prevalence of comorbidities, and healthcare system factors, including underdiagnosis, variable documentation on death certificates, and unequal access to advanced therapies (e.g., ICDs, revascularization). Because CDC WONDER lacks patient‐level diagnostic and treatment data, we cannot separate these contributions; thus, residual confounding and ascertainment bias remain important considerations.

The study highlighted that age‐adjusted mortality rates (AAMRs) were notably higher in the Western and Midwestern United States. These are the regions that have traditionally shown higher rates of smoking, obesity, hypertension, and diabetes (Gurka et al. 2018; Loop et al. 2017), all of which are primary risk factors for ventricular arrhythmias and cardiomyopathy (NIH 2025).

Higher mortality rates were observed in urban populations (Lai et al. 2023) possibly due to higher exposure to environmental toxins, higher comorbidity, and life‐style determinants of physical inactivity and diet (World Health Organization 2025; Hankey et al. 2012) Even with better physical access to healthcare facilities, urban dwellers may be limited by system congestion in the healthcare system (Schull et al. 2002), follow up delay, and socioeconomic differences that limit access to continued, high quality care (Mutono et al. 2022), Initiating more community based preventive interventions, targeted screening, and chronic disease management programs among urban dwellers can help to alleviate some of these differences.

Advancements in the treatment of cardiomyopathy and ventricular arrhythmias have significantly improved clinical outcomes for many individuals. The introduction of implantable cardioverter defibrillators (ICDs) has completely transformed management of cardiomyopathy (CM) in ventricular arrhythmia patients, providing a crucial life‐saving intervention for both primary and secondary prevention (Ezekowitz et al. 2003; Barakat et al. 2017). Antiarrhythmic agents such as amiodarone, sotalol, and beta‐blockers continue to be essential in managing ventricular arrhythmias (Gaine and Calkins 2023), whereas implantable cardiac resynchronization therapy (CRT) is becoming increasingly common in patients with heart failure and refractory arrhythmias (Cho et al. 2012). Looking ahead, innovative therapies are emerging, offering the potential for more effective and personalized treatment options. Gene therapy is under investigation as a potential treatment for genetic arrhythmias, such as Long QT syndrome and arrhythmogenic right ventricular cardiomyopathy (ARVC). The development of CRISPR‐Cas9 gene‐editing technology can offer a method to correct these mutations causing arrhythmias at the molecular level, potentially offering curative therapy for genetic cardiomyopathy (Motta et al. 2017; Trinoskey 2025).

Furthermore, cell‐based therapies, including stem cell therapy, are being explored as potential solutions to regenerate damaged myocardium and to reverse the progression of heart failure. Mesenchymal stem cells (MSCs) (Xu et al. 2024) and induced pluripotent stem cells (iPSCs) (Vo et al. 2024) are being studied for their ability to regenerate fibrotic tissue and stimulate the growth of healthy cardiac muscle (Yamada et al. 2013). New pharmacologic therapies aimed at fibrosis, ion channel dysfunction, and electrical remodeling also are promising to prevent or reduce the incidence of ventricular arrhythmias and sudden cardiac death.

Prior device‐use studies further demonstrate that race and sex intersect. Among 13,034 ICD‐eligible patients hospitalized with heart failure in the Get With The Guidelines–Heart Failure registry from 2005 to 2007, ICD therapy was documented in 33.4% of Black men and 28.2% of Black women, compared with 43.6% of White men (Hernandez et al. 2007). Among Medicare beneficiaries, age‐standardized ICD use increased from 38.0 to 151.7 procedures per 100,000 in Black men and from 18.2 to 77.3 per 100,000 in Black women between 1997 and 2003, with greater absolute use among men (Chan et al. 2009). Later registry data from 2005 to 2009 suggested that racial differences narrowed as ICD use increased, whereas sex differences persisted (Al‐Khatib et al. 2012). These external findings provide plausible health‐system context but cannot explain the mortality pattern in our study. Because our analysis stratified by sex and race separately and did not estimate a race‐by‐sex interaction, we cannot determine the relative contributions of Black men and Black women to the elevated non‐Hispanic Black AAMR; dedicated intersectional analyses are warranted.

The steep decline from 1999 to 2007 temporally overlapped with publication of MADIT II in 2002 and SCD‐HeFT in 2005, followed by rapid expansion of ICD use in U.S. practice (Moss et al. 2002; Bardy et al. 2005; Brown et al. 2008). However, CDC WONDER does not record device implantation, indication, or timing; therefore, the percentage of decedents who had an ICD cannot be calculated, and the joinpoint should not be interpreted as proof that ICD uptake caused the mortality decline. External utilization data are contextual rather than cohort‐specific: U.S. hospitalizations for ICD implantation increased from approximately 5600 in 1990 to more than 108,000 in 2005 (Brown et al. 2008). Concurrent changes in guideline‐directed heart‐failure therapy, CRT, revascularization, diagnostic practices, coding, risk‐factor prevalence, and access to care may also have contributed. Linked mortality, claims, device‐registry, and electronic health record data are needed to test these hypotheses.

## 5 Limitations

Our findings must be understood within the context of multiple limitations, most significantly inherent to the CDC WONDER database's retrospective design. Although the database permits valid trends in mortality, it is unable to report disease prevalence, limiting our capacity to estimate the true burden of cardiomyopathy and ventricular arrhythmia within the population. In addition, potential underreporting or misclassification of these diseases on death certificates, particularly in the early years, was potentially confounded by shifting patterns of diagnoses, varying differences in clinical awareness, and changes in ICD coding guidelines over the study period. Because ICD‐10 codes do not reliably distinguish ischemic from non‐ischemic cardiomyopathy, some overlap and miscoding may have occurred, making etiologic comparisons difficult and potentially biasing temporal trends. Our analysis excluded cases defined by WHO‐assigned ICD‐10 codes, potentially excluding a few diagnoses or including errors, failing to capture the full clinical disease spectrum. Additionally, patient‐level data—including comorbidities, medications, echocardiographic and genetic data, device implantation (ICD/CRT), use of guideline‐directed therapy, insurance coverage, timing of clinical interventions, and lifestyle factors—were unavailable; these variables are critical to understanding the multifactorial interaction between risk factors and outcomes. Finally, socioeconomic and healthcare access variables were not available, which prevented us from investigating the contribution of social determinants of health towards observed mortality inequalities. In addition, sex and race were evaluated as separate strata; without a race‐by‐sex interaction analysis, intersectional differences, including those between Black men and Black women, could not be quantified.

## 6 Conclusion

This study highlights important demographic, racial, and geographic variation in cardiomyopathy and ventricular arrhythmia mortality in the United States during 1999–2024. While overall age‐adjusted mortality rates have declined, men and non‐Hispanic Black populations continue to bear a disproportionately higher burden, with persistently elevated mortality despite advances in therapeutic interventions. The Western and Midwestern regions demonstrated the highest mortality rates, particularly in urban areas due to healthcare congestion and environmental toxins. These findings underscore the need for targeted, population‐specific interventions, such as culturally specific public health programs, early detection programs, and equalized access to sophisticated diagnostic and therapeutic modalities for the elimination of the enduring outcome differences for these high‐risk groups.

## Author Contributions

Conceptualization, Nosheen Pervaiz: Hamza Naveed and Muhammad Junaid Razzak: data curation. Nosheen Pervaiz: Hamza Naveed and Muhammad Hasan: methodology. Muhammad Junaid Razzak: Shaheer Bin Shafiq and Hamza Naveed: writing – original draft. Nosheen Pervaiz: Muhammad Junaid Razzak: Shaheer Bin Shafiq and Muhammad Hasan: writing – review and editing. Hamza Naveed: Muhammad Zia Khan: visualization and tables. Shaheer Bin Shafiq: Muhammad Hasan and Muhammad Junaid Razzak: supervision. Siddharth Agarwal: Zain Ul Abideen Asad: Muhammad Zia Khan: validation and final approval. All authors.

## Funding

The authors have nothing to report.

## Ethics Statement

Institutional review board approval and patient consent were not required because this study used publicly available, de‐identified mortality data from CDC WONDER.

## Conflicts of Interest

The authors declare no conflicts of interest.

## Supporting information


**Table S1:** Ventricular arrhythmias with cardiomyopathy‐related mortalities in adults, stratified by sex and race, in the United States, 1999–2024.
**Table S2:** Ventricular arrhythmias with cardiomyopathy‐related mortalities in adults, stratified by place of death in adults, in the United States, 1999–2024.
**Table S3:** Annual percent change (APC) of ventricular arrhythmias with cardiomyopathy‐related age‐adjusted mortality rates per 100,000 in adults, in the United States, 1999–2024.
**Table S4:** Overall and sex‐stratified ventricular arrhythmias in patients with cardiomyopathy‐related age‐adjusted mortality rates per 100,000 in adults, in the United States, 1999–2024.
**Table S5:** Race‐stratified ventricular arrhythmias in patients with cardiomyopathy‐related age‐adjusted mortality rates per 100,000 in adults, in the United States, 1999–2024.
**Table S6:** Ventricular arrhythmias in patients with cardiomyopathy‐related age‐adjusted mortality rates per 100,000, stratified by states in adults, in the United States, 1999–2020.
**Table S7:** Ventricular arrhythmias in patients with cardiomyopathy‐related age‐adjusted mortality rates per 100,000, stratified by census region in adults, in the United States, 1999–2024.
**Table S8:** Ventricular arrhythmias in patients with cardiomyopathy‐related age‐adjusted mortality rates per 100,000, stratified by urban–rural Classification in adults, in the United States, 1999–2020.
**Table S9:** Subgroup analysis of ventricular arrhythmias in patients with cardiomyopathy‐related mortalities in adults, in the United States, 1999–2024.

## Data Availability

The data that support the findings of this study are publicly available from the Centers for Disease Control and Prevention Wide‐Ranging Online Data for Epidemiologic Research (CDC WONDER) database.
