# Supplementary material for: Understanding the Mortality Trends in Patients With Ventricular Arrhythmias and Cardiomyopathy: A 25‐Year Retrospective Analysis From 1999–2024
Source: Ann Noninvasive Electrocardiol. 2026 Oct 12;31(6):e70234. doi: 10.1111/anec.70234 (PMC13655773; doi:10.1111/anec.70234)
Supplement: Supplementary file 1 [file ANEC-31-e70234-s001.docx]

**Title: Understanding the Mortality Trends in Patients with Ventricular Arrhythmias and Cardiomyopathy: A 25-Year Retrospective Analysis from 1999-2024**

**Running title:** Ventricular Arrhythmias with Cardiomyopathy-related mortality trends in US adults

| **Supplemental Table S1, Ventricular Arrhythmias with Cardiomyopathy-related mortalities in Adults, Stratified by Sex and Race, in the United States, 1999 to 2024** | | | | | | | | |
| --- | --- | --- | --- | --- | --- | --- | --- | --- |
| **Deaths** | | | | | | | | |
| **Year** | **Overall** | **Women** | **Men** | **NH White** | **NH Black** | **NH Others** | **Hispanic** | **Population** |
| **1999** | 4,514 | 1,352 | 3,162 | 3,700 | 531 | 100 | 175 | 180,408,769 |
| **2000** | 4,211 | 1,280 | 2,931 | 3,470 | 492 | 83 | 163 | 181,984,640 |
| **2001** | 3,827 | 1,125 | 2,702 | 3,085 | 469 | 79 | 187 | 184,305,128 |
| **2002** | 3,650 | 1,050 | 2,600 | 2,955 | 426 | 102 | 159 | 186,208,028 |
| **2003** | 3,409 | 946 | 2,463 | 2,729 | 447 | 71 | 161 | 188,090,429 |
| **2004** | 3,107 | 917 | 2,190 | 2,452 | 400 | 90 | 158 | 190,205,384 |
| **2005** | 2,974 | 816 | 2,158 | 2,344 | 390 | 85 | 153 | 192,551,384 |
| **2006** | 2,758 | 784 | 1,974 | 2,182 | 385 | 63 | 121 | 195,019,359 |
| **2007** | 2,642 | 730 | 1,912 | 2,128 | 341 | 60 | 110 | 197,403,777 |
| **2008** | 2,682 | 686 | 1,996 | 2,089 | 384 | 77 | 124 | 199,795,090 |
| **2009** | 2,699 | 739 | 1,960 | 2,124 | 360 | 75 | 133 | 202,107,016 |
| **2010** | 2,728 | 668 | 2,060 | 2,139 | 357 | 96 | 132 | 203,891,983 |
| **2011** | 2,724 | 651 | 2,073 | 2,107 | 396 | 83 | 134 | 206,592,936 |
| **2012** | 2,795 | 732 | 2,063 | 2,115 | 419 | 100 | 158 | 208,826,037 |
| **2013** | 2,870 | 732 | 2,138 | 2,209 | 391 | 94 | 172 | 211,085,314 |
| **2014** | 2,914 | 692 | 2,222 | 2,234 | 419 | 87 | 167 | 213,809,280 |
| **2015** | 3,043 | 781 | 2,262 | 2,347 | 437 | 99 | 155 | 216,553,817 |
| **2016** | 2,904 | 676 | 2,228 | 2,208 | 390 | 104 | 198 | 218,641,417 |
| **2017** | 3,010 | 684 | 2,326 | 2,257 | 428 | 120 | 202 | 221,447,331 |
| **2018** | 3,103 | 790 | 2,313 | 2,309 | 467 | 138 | 184 | 223,311,190 |
| **2019** | 3,106 | 779 | 2,327 | 2,327 | 438 | 136 | 204 | 224,981,167 |
| **2020** | 3,147 | 750 | 2,397 | 2,297 | 471 | 150 | 224 | 226,635,013 |
| **2021** | 3,214 | 776 | 2,438 | 2,333 | 501 | 164 | 210 | 228,238,412 |
| **2022** | 3,163 | 774 | 2,389 | 2,353 | 444 | 137 | 226 | 229,508,599 |
| **2023** | 3,090 | 731 | 2,359 | 2,241 | 463 | 167 | 213 | 231,529,762 |
| **2024** | 2,920 | 729 | 2,191 | 2,086 | 452 | 158 | 215 | 231,529,762 |
| **Total** | **81,204** | **21,370** | **59,834** | **62,820** | **11,098** | **2,718** | **4,438** | **5,394,661,024** |
| NH = non-Hispanic. | | | | | | | | |

| **Supplemental Table S2, Ventricular Arrhythmias with Cardiomyopathy-related mortalities in Adults, Stratified by Place of Death in Adults, in the United States, 1999 to 2024** | | | | |
| --- | --- | --- | --- | --- |
| **Deaths** | | | | |
| **Year** | **Medical Facility** | **Nursing Home/Long-term Care Facility** | **Hospice Facility** | **Home** |
| **1999** | 3,730 | 143 | - | 581 |
| **2000** | 3,407 | 139 | - | 596 |
| **2001** | 3,126 | 137 | - | 524 |
| **2002** | 2,888 | 150 | - | 537 |
| **2003** | 2,694 | 135 | - | 522 |
| **2004** | 2,444 | 134 | - | 470 |
| **2005** | 2,302 | 131 | - | 477 |
| **2006** | 2,208 | 106 | - | 387 |
| **2007** | 2,085 | 99 | 12 | 405 |
| **2008** | 2,069 | 96 | 20 | 407 |
| **2009** | 2,103 | 106 | 26 | 359 |
| **2010** | 2,138 | 122 | 21 | 405 |
| **2011** | 2,175 | 87 | 38 | 385 |
| **2012** | 2,224 | 79 | 36 | 404 |
| **2013** | 2,234 | 104 | 38 | 444 |
| **2014** | 2,264 | 97 | 35 | 464 |
| **2015** | 2,378 | 110 | 54 | 463 |
| **2016** | 2,316 | 86 | 62 | 401 |
| **2017** | 2,338 | 108 | 49 | 470 |
| **2018** | 2,450 | 91 | 52 | 476 |
| **2019** | 2,414 | 92 | 56 | 491 |
| **2020** | 2,352 | 63 | 74 | 590 |
| **2021** | 2,406 | 80 | 63 | 603 |
| **2022** | 2,448 | 80 | 51 | 519 |
| **2023** | 2,393 | 73 | 55 | 528 |
| **2024** | 2,262 | 88 | 63 | 463 |
| **Total** | **63,848** | **2,736** | **805** | **12,371** |

| **Supplemental Table S3 Annual percent change (APC) of Ventricular Arrhythmias with Cardiomyopathy-related Age-Adjusted Mortality Rates per 100,000 in Adults, in the United States, 1999 to 2024** | | |  |
| --- | --- | --- | --- |
| **Year Interval** | **APC (95% CI)** | **P-value** |  |
| **Overall** | | |  |
| 1999-2007 | -7.93(-8.73 to -7.43) | < 0.000001 |  |
| 2007-2022 | -0.66(-0.87 to -0.39) | 0.0024 |  |
| 2022-2024 | -6.87(-9.85 to -3.63) | < 0.000001 |  |
| **Men** | | |  |
| 1999-2006 | -8.54(-10.16 to -7.58) | < 0.000001 |  |
| 2006-2022 | -1.03(-7.74 to 0.64) | 0.057189 |  |
| 2022-2024 | -5.08(-8.86 to -1.16) | < 0.000001 |  |
| **Women** | | |  |
| 1999-2008 | -8.62(-10.54 to -7.43) | < 0.000001 |  |
| 2008-2024 | -0.88(-1.58 to -0.11) | 0.028794 |  |
| **NH White** | | |  |
| 1999-2006 | -8.74(-10.12 to -7.67) | < 0.000001 |  |
| 2006-2024 | -1.07(-1.40 to -0.69) | < 0.000001 |  |
| **NH Black** | | |  |
| 1999-2007 | -6.67(-11.18 to -4.85) | < 0.000001 |  |
| 2007-2024 | -0.88(-1.57 to 0.00) | 0.05159 |  |
| **NH Others** | | |  |
| 1999-2024 | -2.22(-3.29 to -0.92) | 0.002 |  |
| **Hispanic** | | |  |
| 1999-2007 | -10.24(-19.74 to -6.60) | < 0.000001 |  |
| 2007-2024 | -1.00(-2.24 to 0.90) | 0.19836 |  |
| **Rural areas** | | |  |
| 1999-2006 | -7.70(-10.06 to -6.15) | < 0.000001 |  |
| 2006-2020 | -0.38(-1.07 to 0.47) | 0.353929 |  |
| **Urban areas** | | |  |
| 1999-2007 | -8.15(-8.39 to -7.91) | < 0.000001 |  |
| 2007-2020 | -0.83(-0.97 to -0.68) | < 0.000001 |  |
| **Northeast region** | | |  |
| 1999-2006 | -9.39(-11.68 to -7.63) | < 0.000001 |  |
| 2006-2024 | -0.48(-1.01 to 0.10) | 0.10238 |  |
| **South region** | | |  |
| 1999-2007 | -8.15(-9.30 to -7.26) | < 0.000001 |  |
| 2007-2024 | -1.12(-1.43 to -0.77) | < 0.000001 |  |
| **Midwest region** | | |  |
| 1999-2024 | -2.52(-3.49 to -1.59) | < 0.000001 |  |
| **West region** | | |  |
| 1999-2006 | -8.08(-11.48 to -6.19) | < 0.000001 |  |
| 2006-2024 | -1.67(-2.24 to -1.04) | 0.0012 |  |
| APC = annual percent change; NH = non-Hispanic; * Indicates that the annual percentage change (APC) is significantly different from zero at α = 0.05. AAMR = age-adjusted mortality rate. The data for urbanization was only available till 2020 in the CDC Wonder Database. | | |  |
|  |  |  |  |

| **Supplemental Table S4 Overall and Sex‐Stratified Ventricular Arrhythmias in patients with Cardiomyopathy-related Age-Adjusted Mortality Rates per 100,000 in adults, in the United States, 1999 to 2024** | | | |
| --- | --- | --- | --- |
| **Age-Adjusted Rate (95% CI)** | | | |
| **Year** | **Men** | **Women** | **Overall** |
| **1999** | 4.25 (4.09 - 4.40) | 1.33 (1.26 - 1.40) | 2.54 (2.47 - 2.62) |
| **2000** | 3.91 (3.76 - 4.05) | 1.23 (1.16 - 1.30) | 2.36 (2.29 - 2.43) |
| **2001** | 3.53 (3.39 - 3.66) | 1.09 (1.03 - 1.15) | 2.11 (2.04 - 2.17) |
| **2002** | 3.35 (3.22 - 3.48) | 1.00 (0.94 - 1.06) | 1.95 (1.89 - 2.02) |
| **2003** | 3.09 (2.96 - 3.21) | 0.90 (0.84 - 0.96) | 1.80 (1.74 - 1.86) |
| **2004** | 2.70 (2.59 - 2.82) | 0.83 (0.78 - 0.89) | 1.62 (1.57 - 1.68) |
| **2005** | 2.58 (2.47 - 2.69) | 0.74 (0.69 - 0.79) | 1.53 (1.47 - 1.58) |
| **2006** | 2.33 (2.22 - 2.43) | 0.70 (0.65 - 0.74) | 1.38 (1.33 - 1.43) |
| **2007** | 2.22 (2.12 - 2.32) | 0.64 (0.59 - 0.68) | 1.32 (1.27 - 1.37) |
| **2008** | 2.24 (2.14 - 2.34) | 0.59 (0.54 - 0.63) | 1.30 (1.25 - 1.35) |
| **2009** | 2.14 (2.05 - 2.24) | 0.64 (0.59 - 0.69) | 1.27 (1.22 - 1.32) |
| **2010** | 2.23 (2.13 - 2.33) | 0.55 (0.50 - 0.59) | 1.31 (1.26 - 1.36) |
| **2011** | 2.17 (2.08 - 2.27) | 0.53 (0.49 - 0.57) | 1.27 (1.23 - 1.32) |
| **2012** | 2.11 (2.01 - 2.20) | 0.58 (0.54 - 0.63) | 1.26 (1.21 - 1.30) |
| **2013** | 2.14 (2.05 - 2.23) | 0.58 (0.53 - 0.62) | 1.25 (1.20 - 1.29) |
| **2014** | 2.14 (2.05 - 2.23) | 0.51 (0.47 - 0.55) | 1.24 (1.20 - 1.29) |
| **2015** | 2.12 (2.03 - 2.21) | 0.60 (0.56 - 0.64) | 1.28 (1.23 - 1.33) |
| **2016** | 2.05 (1.96 - 2.14) | 0.48 (0.44 - 0.52) | 1.20 (1.16 - 1.25) |
| **2017** | 2.07 (1.99 - 2.16) | 0.49 (0.46 - 0.53) | 1.21 (1.16 - 1.25) |
| **2018** | 1.99 (1.91 - 2.07) | 0.55 (0.51 - 0.59) | 1.19 (1.15 - 1.23) |
| **2019** | 1.97 (1.89 - 2.05) | 0.57 (0.53 - 0.61) | 1.20 (1.15 - 1.24) |
| **2020** | 1.98 (1.90 - 2.06) | 0.49 (0.45 - 0.53) | 1.17 (1.13 - 1.21) |
| **2021** | 2.01 (1.93 - 2.09) | 0.57 (0.53 - 0.61) | 1.20 (1.16 - 1.24) |
| **2022** | 1.95 (1.87 - 2.03) | 0.53 (0.49 - 0.57) | 1.16 (1.12 - 1.20) |
| **2023** | 1.87 (1.79 - 1.95) | 0.51 (0.47 - 0.54) | 1.11 (1.07 - 1.15) |
| **2024** | 1.74 (1.66 - 1.81) | 0.48 (0.44 - 0.52) | 1.01 (0.98 - 1.05) |
| **Total** | **2.42 (2.32 - 2.52)** | **0.68 (0.63 - 0.73)** | **1.43 (1.38 - 1.48)** |

| **Supplemental Table S5, Race‐Stratified Ventricular Arrhythmias in patients with Cardiomyopathy-related Age-Adjusted Mortality Rates per 100,000 in adults, in the United States, 1999 to 2024** | | | | |
| --- | --- | --- | --- | --- |
| **Age-Adjusted Rate (95% CI)** | | | | |
| **Year** | **NH White** | **NH Black** | **NH Others** | **Hispanic** |
| **1999** | 2.52 (2.44 - 2.60) | 3.27 (2.99 - 3.55) | 1.83 (1.46 - 2.20) | 1.94 (1.64 - 2.24) |
| **2000** | 2.35 (2.27 - 2.42) | 3.00 (2.73 - 3.27) | 1.53 (1.20 - 1.91) | 1.63 (1.37 - 1.89) |
| **2001** | 2.07 (2.00 - 2.15) | 2.77 (2.51 - 3.02) | 1.40 (1.09 - 1.76) | 1.80 (1.53 - 2.07) |
| **2002** | 1.95 (1.88 - 2.02) | 2.49 (2.25 - 2.73) | 1.57 (1.25 - 1.90) | 1.43 (1.19 - 1.66) |
| **2003** | 1.79 (1.72 - 1.86) | 2.52 (2.28 - 2.76) | 0.96 (0.74 - 1.23) | 1.36 (1.14 - 1.59) |
| **2004** | 1.59 (1.53 - 1.66) | 2.20 (1.98 - 2.43) | 1.19 (0.95 - 1.48) | 1.27 (1.06 - 1.47) |
| **2005** | 1.51 (1.45 - 1.57) | 2.08 (1.87 - 2.30) | 1.11 (0.88 - 1.39) | 1.14 (0.95 - 1.33) |
| **2006** | 1.35 (1.29 - 1.40) | 2.07 (1.85 - 2.28) | 0.78 (0.59 - 1.01) | 0.86 (0.70 - 1.02) |
| **2007** | 1.30 (1.25 - 1.36) | 1.75 (1.56 - 1.94) | 0.70 (0.53 - 0.91) | 0.78 (0.63 - 0.94) |
| **2008** | 1.25 (1.20 - 1.31) | 1.94 (1.74 - 2.14) | 0.92 (0.72 - 1.17) | 0.81 (0.66 - 0.96) |
| **2009** | 1.31 (1.25 - 1.36) | 1.73 (1.55 - 1.92) | 0.81 (0.63 - 1.03) | 0.77 (0.63 - 0.91) |
| **2010** | 1.25 (1.19 - 1.30) | 1.72 (1.54 - 1.91) | 1.00 (0.80 - 1.23) | 0.81 (0.67 - 0.96) |
| **2011** | 1.23 (1.17 - 1.28) | 1.84 (1.65 - 2.02) | 0.79 (0.62 - 0.99) | 0.73 (0.60 - 0.86) |
| **2012** | 1.23 (1.17 - 1.28) | 1.84 (1.66 - 2.03) | 0.93 (0.74 - 1.11) | 0.86 (0.72 - 1.00) |
| **2013** | 1.27 (1.22 - 1.33) | 1.73 (1.55 - 1.90) | 0.79 (0.63 - 0.97) | 0.89 (0.75 - 1.03) |
| **2014** | 1.24 (1.19 - 1.29) | 1.80 (1.62 - 1.98) | 0.72 (0.57 - 0.89) | 0.79 (0.66 - 0.92) |
| **2015** | 1.29 (1.24 - 1.35) | 1.78 (1.61 - 1.96) | 0.76 (0.62 - 0.93) | 0.68 (0.57 - 0.79) |
| **2016** | 1.16 (1.11 - 1.21) | 1.56 (1.40 - 1.72) | 0.76 (0.61 - 0.91) | 0.84 (0.72 - 0.96) |
| **2017** | 1.19 (1.14 - 1.24) | 1.65 (1.49 - 1.81) | 0.83 (0.68 - 0.98) | 0.80 (0.69 - 0.92) |
| **2018** | 1.18 (1.13 - 1.23) | 1.76 (1.60 - 1.93) | 0.92 (0.76 - 1.08) | 0.69 (0.59 - 0.80) |
| **2019** | 1.22 (1.17 - 1.27) | 1.58 (1.43 - 1.73) | 0.86 (0.71 - 1.01) | 0.81 (0.69 - 0.92) |
| **2020** | 1.15 (1.10 - 1.19) | 1.68 (1.53 - 1.84) | 0.92 (0.77 - 1.07) | 0.80 (0.70 - 0.91) |
| **2021** | 1.22 (1.17 - 1.27) | 1.79 (1.63 - 1.95) | 0.89 (0.75 - 1.03) | 0.74 (0.64 - 0.85) |
| **2022** | 1.17 (1.12 - 1.22) | 1.53 (1.38 - 1.68) | 0.71 (0.59 - 0.82) | 0.75 (0.65 - 0.85) |
| **2023** | 1.13 (1.08 - 1.17) | 1.60 (1.45 - 1.75) | 0.84 (0.71 - 0.96) | 0.66 (0.57 - 0.75) |
| **2024** | 1.01 (0.96 - 1.05) | 1.54 (1.40 - 1.69) | 0.78 (0.65 - 0.90) | 0.68 (0.58 - 0.77) |
| **Total** | **1.42 (1.36 - 1.48)** | **1.97 (1.78 - 2.16)** | **0.97 (0.78 - 1.19)** | **0.97 (0.82 - 1.13)** |
| NH = non-Hispanic. | | | | |

| **Supplemental Table S6, Ventricular Arrhythmias in patients with Cardiomyopathy-related Age-Adjusted Mortality Rates per 100,000, Stratified by States in adults, in the United States, 1999 to 2020** | |
| --- | --- |
| **State** | **Age-Adjusted Rate (95% CI)** |
| Alabama | 1.26 (1.18 - 1.34) |
| Alaska | 1.55 (1.25 - 1.85) |
| Arizona | 1.38 (1.31 - 1.45) |
| Arkansas | 1.25 (1.15 - 1.35) |
| California | 1.63 (1.59 - 1.66) |
| Colorado | 1.07 (0.99 - 1.15) |
| Connecticut | 1.07 (0.99 - 1.16) |
| Delaware | 2.20 (1.95 - 2.44) |
| District of Columbia | 1.51 (1.24 - 1.77) |
| Florida | 1.47 (1.43 - 1.51) |
| Georgia | 1.25 (1.19 - 1.32) |
| Hawaii | 1.85 (1.67 - 2.03) |
| Idaho | 1.70 (1.53 - 1.87) |
| Illinois | 1.19 (1.14 - 1.24) |
| Indiana | 2.11 (2.02 - 2.20) |
| Iowa | 1.33 (1.23 - 1.43) |
| Kansas | 1.34 (1.23 - 1.45) |
| Kentucky | 1.24 (1.15 - 1.32) |
| Louisiana | 1.12 (1.04 - 1.20) |
| Maine | 1.32 (1.18 - 1.46) |
| Maryland | 1.49 (1.41 - 1.57) |
| Massachusetts | 1.00 (0.94 - 1.06) |
| Michigan | 1.39 (1.33 - 1.45) |
| Minnesota | 1.18 (1.11 - 1.26) |
| Mississippi | 1.28 (1.17 - 1.38) |
| Missouri | 1.73 (1.65 - 1.82) |
| Montana | 0.99 (0.84 - 1.15) |
| Nebraska | 1.09 (0.97 - 1.21) |
| Nevada | 1.08 (0.98 - 1.19) |
| New Hampshire | 1.20 (1.05 - 1.35) |
| New Jersey | 1.49 (1.43 - 1.55) |
| New Mexico | 0.90 (0.80 - 1.01) |
| New York | 0.83 (0.80 - 0.86) |
| North Carolina | 1.81 (1.74 - 1.88) |
| North Dakota | 1.29 (1.08 - 1.51) |
| Ohio | 1.86 (1.80 - 1.92) |
| Oklahoma | 1.35 (1.25 - 1.44) |
| Oregon | 1.42 (1.33 - 1.52) |
| Pennsylvania | 1.69 (1.63 - 1.74) |
| Rhode Island | 1.24 (1.07 - 1.40) |
| South Carolina | 1.79 (1.69 - 1.89) |
| South Dakota | 1.09 (0.91 - 1.27) |
| Tennessee | 1.98 (1.89 - 2.07) |
| Texas | 1.51 (1.47 - 1.56) |
| Utah | 1.30 (1.18 - 1.43) |
| Vermont | 1.32 (1.10 - 1.54) |
| Virginia | 1.34 (1.27 - 1.41) |
| Washington | 2.11 (2.02 - 2.20) |
| West Virginia | 1.36 (1.23 - 1.48) |
| Wisconsin | 1.39 (1.32 - 1.47) |
| Wyoming | 1.10 (0.88 - 1.36) |

| **Supplemental Table S7, Ventricular Arrhythmias in patients with Cardiomyopathy-related Age-Adjusted Mortality Rates per 100,000, Stratified by Census Region in adults, in the United States, 1999 to 2024** | | | | |
| --- | --- | --- | --- | --- |
|  | **Census Region: NorthEast** | **Census Region: Midwest** | **Census Region:  South** | **Census Region:  West** |
| **Year** | **Age-Adjusted Rate (95% CI)** | **Age-Adjusted Rate (95% CI)** | **Age-Adjusted Rate (95% CI)** | **Age-Adjusted Rate (95% CI)** |
| **1999** | 2.21 (2.06 - 2.37) | 2.54 (2.39 - 2.69) | 2.63 (2.50 - 2.75) | 2.72 (2.55 - 2.89) |
| **2000** | 1.93 (1.79 - 2.07) | 2.31 (2.16 - 2.45) | 2.45 (2.33 - 2.57) | 2.62 (2.46 - 2.79) |
| **2001** | 1.81 (1.67 - 1.95) | 1.98 (1.85 - 2.12) | 2.27 (2.16 - 2.39) | 2.18 (2.03 - 2.33) |
| **2002** | 1.59 (1.46 - 1.71) | 2.10 (1.97 - 2.24) | 2.07 (1.96 - 2.18) | 2.05 (1.91 - 2.20) |
| **2003** | 1.42 (1.30 - 1.54) | 1.87 (1.74 - 2.00) | 1.90 (1.80 - 2.01) | 1.91 (1.78 - 2.05) |
| **2004** | 1.29 (1.17 - 1.40) | 1.69 (1.57 - 1.81) | 1.61 (1.52 - 1.71) | 1.88 (1.75 - 2.02) |
| **2005** | 1.23 (1.12 - 1.34) | 1.52 (1.41 - 1.64) | 1.59 (1.49 - 1.68) | 1.70 (1.57 - 1.82) |
| **2006** | 1.19 (1.08 - 1.30) | 1.46 (1.35 - 1.58) | 1.44 (1.35 - 1.53) | 1.42 (1.30 - 1.53) |
| **2007** | 1.09 (0.98 - 1.19) | 1.41 (1.31 - 1.52) | 1.32 (1.23 - 1.40) | 1.37 (1.25 - 1.48) |
| **2008** | 0.97 (0.87 - 1.07) | 1.29 (1.19 - 1.40) | 1.37 (1.29 - 1.46) | 1.51 (1.39 - 1.62) |
| **2009** | 1.03 (0.93 - 1.13) | 1.34 (1.24 - 1.45) | 1.31 (1.23 - 1.39) | 1.39 (1.28 - 1.50) |
| **2010** | 1.02 (0.93 - 1.12) | 1.30 (1.20 - 1.40) | 1.25 (1.17 - 1.33) | 1.50 (1.39 - 1.61) |
| **2011** | 1.10 (1.00 - 1.20) | 1.25 (1.15 - 1.35) | 1.24 (1.16 - 1.31) | 1.39 (1.28 - 1.49) |
| **2012** | 1.07 (0.97 - 1.17) | 1.23 (1.13 - 1.33) | 1.29 (1.21 - 1.37) | 1.40 (1.29 - 1.51) |
| **2013** | 1.08 (0.98 - 1.18) | 1.28 (1.18 - 1.38) | 1.31 (1.23 - 1.38) | 1.26 (1.16 - 1.36) |
| **2014** | 1.09 (0.99 - 1.19) | 1.35 (1.25 - 1.45) | 1.23 (1.15 - 1.30) | 1.27 (1.18 - 1.37) |
| **2015** | 1.07 (0.97 - 1.16) | 1.28 (1.18 - 1.38) | 1.32 (1.25 - 1.40) | 1.26 (1.16 - 1.36) |
| **2016** | 0.97 (0.87 - 1.06) | 1.18 (1.08 - 1.27) | 1.21 (1.14 - 1.29) | 1.34 (1.24 - 1.44) |
| **2017** | 1.00 (0.91 - 1.10) | 1.20 (1.11 - 1.30) | 1.23 (1.16 - 1.30) | 1.26 (1.17 - 1.35) |
| **2018** | 0.96 (0.87 - 1.05) | 1.31 (1.21 - 1.41) | 1.16 (1.09 - 1.23) | 1.29 (1.20 - 1.39) |
| **2019** | 1.04 (0.95 - 1.14) | 1.34 (1.24 - 1.44) | 1.13 (1.07 - 1.20) | 1.23 (1.14 - 1.32) |
| **2020** | 1.01 (0.92 - 1.10) | 1.35 (1.25 - 1.45) | 1.15 (1.09 - 1.22) | 1.21 (1.12 - 1.30) |
| **2021** | 1.12 (1.02 - 1.21) | 1.32 (1.22 - 1.42) | 1.22 (1.15 - 1.29) | 1.27 (1.18 - 1.37) |
| **2022** | 1.13 (1.04 - 1.23) | 1.19 (1.10 - 1.28) | 1.13 (1.06 - 1.19) | 1.16 (1.07 - 1.25) |
| **2023** | 0.96 (0.87 - 1.04) | 1.25 (1.15 - 1.34) | 1.11 (1.05 - 1.18) | 1.12 (1.03 - 1.20) |
| **2024** | 0.90 (0.82 - 0.99) | 1.17 (1.08 - 1.26) | 1.08 (1.02 - 1.15) | 0.98 (0.90 - 1.06) |
| **Total** | **1.20 (1.10 - 1.31)** | **1.48 (1.37 - 1.59)** | **1.46 (1.38 - 1.55)** | **1.53 (1.41 - 1.64)** |

| **Supplemental Table S8, Ventricular Arrhythmias in patients with Cardiomyopathy-related Age-Adjusted Mortality Rates per 100,000, Stratified by Urban-Rural Classification in adults, in the United States, 1999 to 2020** | | |  |
| --- | --- | --- | --- |
| **Age-Adjusted Rate (95% CI)** | | |  |
| **Year** | **Urban** | **Rural** |  |
| **1999** | 2.60 (2.52 - 2.68) | 2.32 (2.15 - 2.49) |  |
| **2000** | 2.40 (2.32 - 2.48) | 2.08 (1.93 - 2.24) |  |
| **2001** | 2.16 (2.09 - 2.24) | 1.78 (1.63 - 1.92) |  |
| **2002** | 2.01 (1.94 - 2.08) | 1.79 (1.65 - 1.93) |  |
| **2003** | 1.83 (1.77 - 1.90) | 1.63 (1.50 - 1.77) |  |
| **2004** | 1.66 (1.59 - 1.72) | 1.52 (1.39 - 1.65) |  |
| **2005** | 1.55 (1.49 - 1.61) | 1.46 (1.34 - 1.59) |  |
| **2006** | 1.41 (1.36 - 1.47) | 1.24 (1.12 - 1.36) |  |
| **2007** | 1.32 (1.26 - 1.37) | 1.30 (1.18 - 1.42) |  |
| **2008** | 1.30 (1.24 - 1.36) | 1.33 (1.21 - 1.45) |  |
| **2009** | 1.29 (1.23 - 1.34) | 1.24 (1.13 - 1.36) |  |
| **2010** | 1.30 (1.24 - 1.35) | 1.29 (1.17 - 1.40) |  |
| **2011** | 1.25 (1.20 - 1.31) | 1.23 (1.12 - 1.34) |  |
| **2012** | 1.29 (1.24 - 1.34) | 1.12 (1.01 - 1.23) |  |
| **2013** | 1.26 (1.21 - 1.31) | 1.20 (1.09 - 1.31) |  |
| **2014** | 1.24 (1.19 - 1.29) | 1.32 (1.21 - 1.44) |  |
| **2015** | 1.25 (1.20 - 1.30) | 1.36 (1.24 - 1.47) |  |
| **2016** | 1.20 (1.15 - 1.25) | 1.25 (1.13 - 1.36) |  |
| **2017** | 1.21 (1.16 - 1.26) | 1.20 (1.09 - 1.31) |  |
| **2018** | 1.19 (1.14 - 1.23) | 1.19 (1.08 - 1.30) |  |
| **2019** | 1.18 (1.14 - 1.23) | 1.22 (1.11 - 1.33) |  |
| **2020** | 1.17 (1.12 - 1.21) | 1.24 (1.13 - 1.35) |  |
| **Total** | **1.45 (1.44 - 1.47)** | **1.40 (1.37 - 1.42)** |  |
| The data for urbanization was only available till 2020 in the CDC Wonder Database. | | |  |
|  |  |  |  |

| **Supplemental Table S9, Subgroup Analysis of Ventricular Arrhythmias in patients with Cardiomyopathy-related mortalities in adults, in the United States, 1999 to 2024** | | | | | | | | | | | |
| --- | --- | --- | --- | --- | --- | --- | --- | --- | --- | --- | --- |
| **Year** | **I25.5**  (Ischemic cardiomyopathy)) | **I42.0**  (Dilated cardiomyopathy) | **I42.1** (Obstructive hypertrophic cardiomyopathy) | **I42.2**  (Other hypertrophic cardiomyopathy) | **I42.3**  (Endomyocardial (eosinophilic) disease) | **I42.4**  (Endocardial fibroelastosis) | **I42.5**  (Other restrictive cardiomyopathy) | **I42.6**  (Alcoholic cardiomyopathy) | **I42.7**  (Cardiomyopathy due to drugs and other external agents) | **I42.8**  (Other cardiomyopathies) | **I42.9**  (Cardiomyopathy, Mixed or Unspecified) |
| **Deaths** | | | | | | | | | | | |
| 1999 | 1977 | 680 | 19 | 38 | - | - | - | 23 | - | - | 1803 |
| 2000 | 1837 | 575 | 18 | 44 | - | - | - | 15 | - | - | 1739 |
| 2001 | 1626 | 524 | 13 | 31 | - | - | - | 24 | - | - | 1615 |
| 2002 | 1576 | 508 | 11 | 30 | - | - | - | 10 | - | - | 1518 |
| 2003 | 1474 | 442 | 13 | 34 | - | - | - | 12 | - | - | 1439 |
| 2004 | 1400 | 458 | 12 | 27 | - | - | - | 15 | - | - | 1198 |
| 2005 | 1323 | 412 | - | 29 | - | - | - | - | - | - | 1197 |
| 2006 | 1200 | 374 | - | 23 | - | - | - | 21 | - | - | 1135 |
| 2007 | 1255 | 291 | - | 24 | - | - | - | 34 | - | - | 1031 |
| 2008 | 1232 | 326 | - | 28 | - | - | - | 22 | - | - | 1072 |
| 2009 | 1252 | 322 | - | 22 | - | - | - | 28 | - | - | 1069 |
| 2010 | 1318 | 287 | - | 21 | - | - | - | 30 | - | - | 1069 |
| 2011 | 1320 | 245 | - | 28 | - | - | - | 26 | - | - | 1101 |
| 2012 | 1346 | 225 | - | 19 | - | - | - | 29 | - | - | 1167 |
| 2013 | 1378 | 206 | - | 24 | - | - | - | 30 | - | - | 1223 |
| 2014 | 1424 | 225 | - | 25 | - | - | - | 25 | - | - | 1211 |
| 2015 | 1460 | 245 | - | 24 | - | - | - | 28 | - | - | 1280 |
| 2016 | 1440 | 217 | - | 28 | - | - | - | 24 | - | - | 1191 |
| 2017 | 1510 | 215 | 11 | 34 | - | - | - | 27 | - | 12 | 1210 |
| 2018 | 1511 | 239 | 11 | 37 | - | - | 12 | 28 | - | 16 | 1268 |
| 2019 | 1540 | 228 |  | 38 | - | - | - | 24 | 10 | 10 | 1256 |
| 2020 | 1557 | 254 | 16 | 36 | - | - | - | 29 | - | 20 | 1249 |
| 2021 | 1549 | 197 | 12 | 43 | - | - | - | 35 | - | 18 | 1358 |
| 2022 | 1513 | 229 | - | 45 | - | - | - | 26 | - | 15 | 1334 |
| 2023 | 1546 | 209 | 11 | 42 | - | - | - | 28 | - | 23 | 1237 |
| 2024 | 1408 | 219 | 16 | 49 | - | - | - | 14 | - | 21 | 1203 |
| **Total** | **37,972** | **8352** | **163** | **823** | **-** | **28** | **110** | **607** | **59** | **135** | **33,173** |
